# Orthodontic Microscrew for Ectopic Maxillary Canine Extraction in an Adolescent: A Case Report

**DOI:** 10.7759/cureus.113468

**Published:** 2026-07-27

**Authors:** Nadia Verónica González Carrillo, Fabiola Hernández Girón, Silvia Tavira Fernández, Cesar Esquivel Chirino

**Affiliations:** 1 Orthodontics, División de Estudios de Posgrado e Investigación, Facultad de Odontología, Universidad Nacional Autónoma de México, Mexico City, MEX; 2 Dentistry, Facultad de Odontología, Universidad Nacional Autónoma de México, Mexico City, MEX

**Keywords:** cone-beam computed tomography, maxillary canine impaction, orthodontic microscrew, temporary anchorage device, transitional prosthesis

## Abstract

Extraction of a permanent maxillary canine in a skeletally immature patient creates a rehabilitative interval during which definitive implant therapy is contraindicated and conventional alternatives have well-known limitations. This case report describes the use of a titanium orthodontic microscrew as a transitional prosthetic support in this clinical situation. A 13-year-old female patient was referred with an impacted right maxillary canine in a severely ectopic position adjacent to the floor of the maxillary sinus. Orthodontic traction was considered unfeasible based on the tooth position, axial inclination, and the presence of root dilaceration in the adjacent first premolar. Following extraction and orthodontic recovery of the edentulous space, cone-beam computed tomography (CBCT)-based digital planning and a custom surgical guide were used to place a 1.36 × 12 mm titanium microscrew vertically in the canine site. A provisional polymethyl methacrylate crown was designed by computer-aided design and computer-aided manufacturing (CAD/CAM) and fabricated by 3D printing, then delivered under a non-functional immediate loading protocol. After 12 months of clinical and radiographic follow-up, the soft tissues surrounding the microscrew were healthy, and no biologic or mechanical complications were recorded. A control CBCT at 12 months showed no apparent change in the alveolar contour at the treated site.

## Introduction

The maxillary canine is the second most frequently impacted tooth after the third molar, with a reported prevalence ranging from 1% to 4% and a higher occurrence in females [1,2]. Its long, complex eruption pathway accounts for this susceptibility, and accurate 3D localization is essential for planning its management [3]. Cone-beam computed tomography (CBCT) has become the imaging modality of choice for evaluating the position of the impacted tooth, the proximity of adjacent roots, and the presence of complications such as root resorption [4].

Beyond its esthetic prominence, the maxillary canine is widely regarded as a cornerstone of the dental arch: its long root and strategic position contribute to arch form, and in a mutually protected (canine-guided) occlusion, it disoccludes the posterior teeth during lateral excursions, reducing posterior lateral contacts [5]. The functional importance of this guidance is directly relevant to the present case, because any device intended to occupy the canine position must be protected from the lateral and shearing forces normally borne by a natural canine.

Surgical exposure followed by orthodontic traction is the treatment of choice in most cases. The feasibility and likely difficulty of traction can be estimated preoperatively using recognized radiographic features, including the mesiodistal position or sector of the canine crown and its angulation to the midline [6]. However, severe ectopia, extreme angulation, ankylosis, or close proximity to anatomic structures such as the maxillary sinus may render traction unfeasible or carry an unacceptable biologic risk [7]. When extraction is indicated for an adolescent patient, the rehabilitative phase must accommodate ongoing craniofacial growth. Definitive osseointegrated implants are generally deferred until skeletal maturity is reached, since their ankylosed behavior does not follow alveolar development and may result in progressive infraocclusion over time [8,9].

When extraction is chosen, several transitional strategies may be considered before definitive rehabilitation, each with recognized limitations in growing patients: removable prostheses (poor adolescent tolerance, no physiologic load transfer); resin-bonded/adhesive restorations (commit otherwise intact adjacent teeth); orthodontic space closure by canine or premolar substitution (dependent on occlusion and arch relationships); and autotransplantation (contingent on donor-root stage, technique-sensitive, with ankylosis/resorption risk). Against this background, a microscrew-supported provisional offers a minimally invasive and reversible option that preserves both space and adjacent tooth integrity [10-14].

Although a microscrew is retained mechanically rather than through osseointegration, it is particularly susceptible to lateral and shearing loads. This consideration is central to the canine region, where guidance-related lateral forces are greatest, and it directly informed the decision to deliver the provisional restoration under a strict non-functional loading protocol. This report describes the interdisciplinary management of an adolescent patient with a severely ectopic maxillary canine deemed unsuitable for orthodontic traction, in whom extraction was followed by placement of a titanium orthodontic microscrew as transitional prosthetic support. Clinical and radiographic findings during a 12-month follow-up period are presented.

## Case presentation

**Figure 1:**
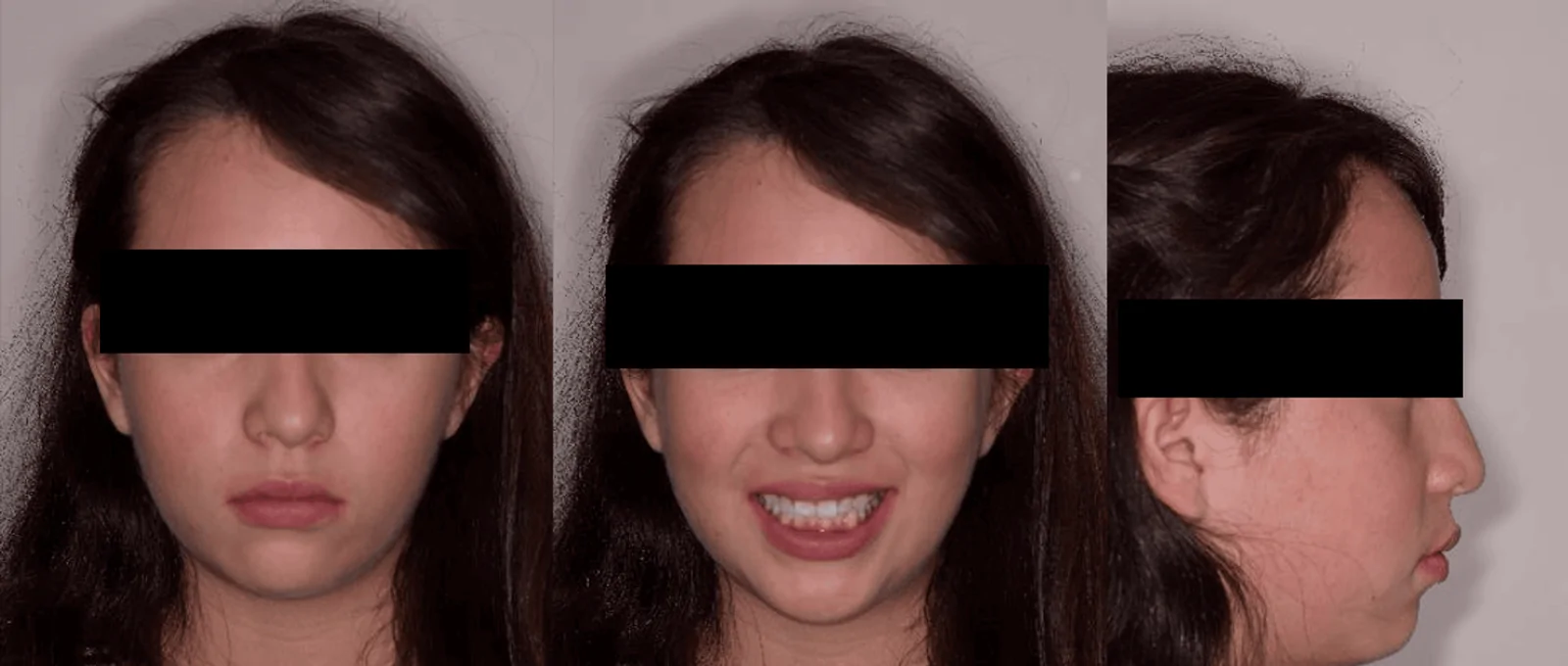
Pre-treatment extraoral photographs Frontal and profile views showing the initial facial appearance of the patient before treatment.

A 13-year-old female patient was referred to the Postgraduate Studies and Research Division, Faculty of Dentistry, National Autonomous University of Mexico (UNAM), for orthodontic evaluation. The medical history was unremarkable, with no relevant systemic conditions, no known drug allergies, and no previous surgery involving the anterior maxilla. Extraoral examination showed a convex facial profile, preserved vertical proportions, and no clinically evident mandibular functional alteration (Figure 1).

**Figure 2:**
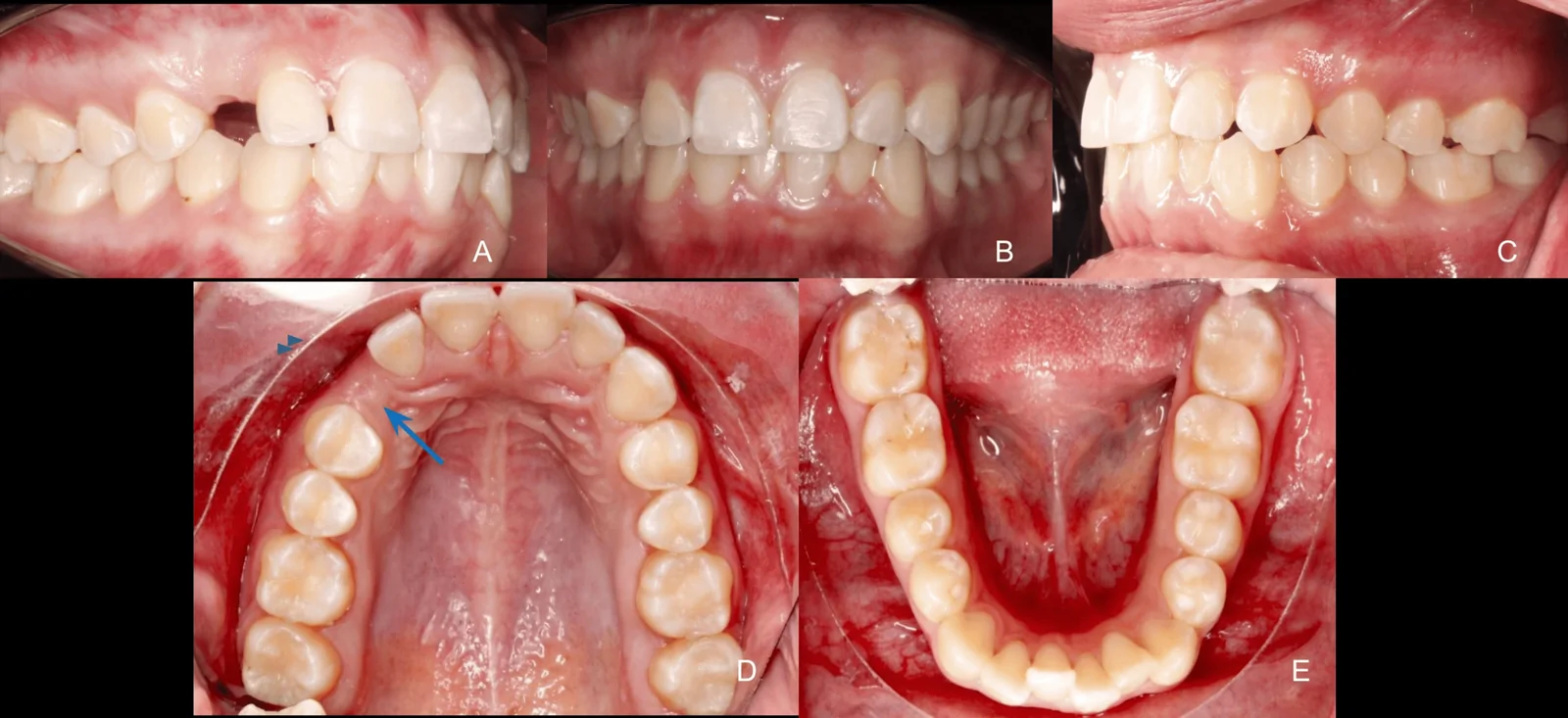
Pre-treatment intraoral photographs A: Right lateral view, B: Frontal view, C: Left lateral view, D: Maxillary occlusal view (blue arrow marks the edentulous area of the retained maxillary canine (13#),  E: Vertical overbite, F: Mandibular occlusal view

Intraoral examination revealed persistence of the upper right primary canine and clinical absence of tooth 13. A class I canine relationship was identified on the left side. The right side could not be classified because of the absence of the permanent canine. Both permanent second molars had erupted fully. Periodontal probing of the anterior region showed no signs of active inflammation or clinical attachment loss at the time of the orthodontic assessment (Figure 2). The clinical absence of tooth 13 was differentiated among impaction, agenesis, previous extraction, and transmigration. Persistence of the primary canine, together with CBCT localization of an unerupted, severely ectopic permanent canine, confirmed impaction and excluded agenesis and prior extraction.

**Figure 3:**
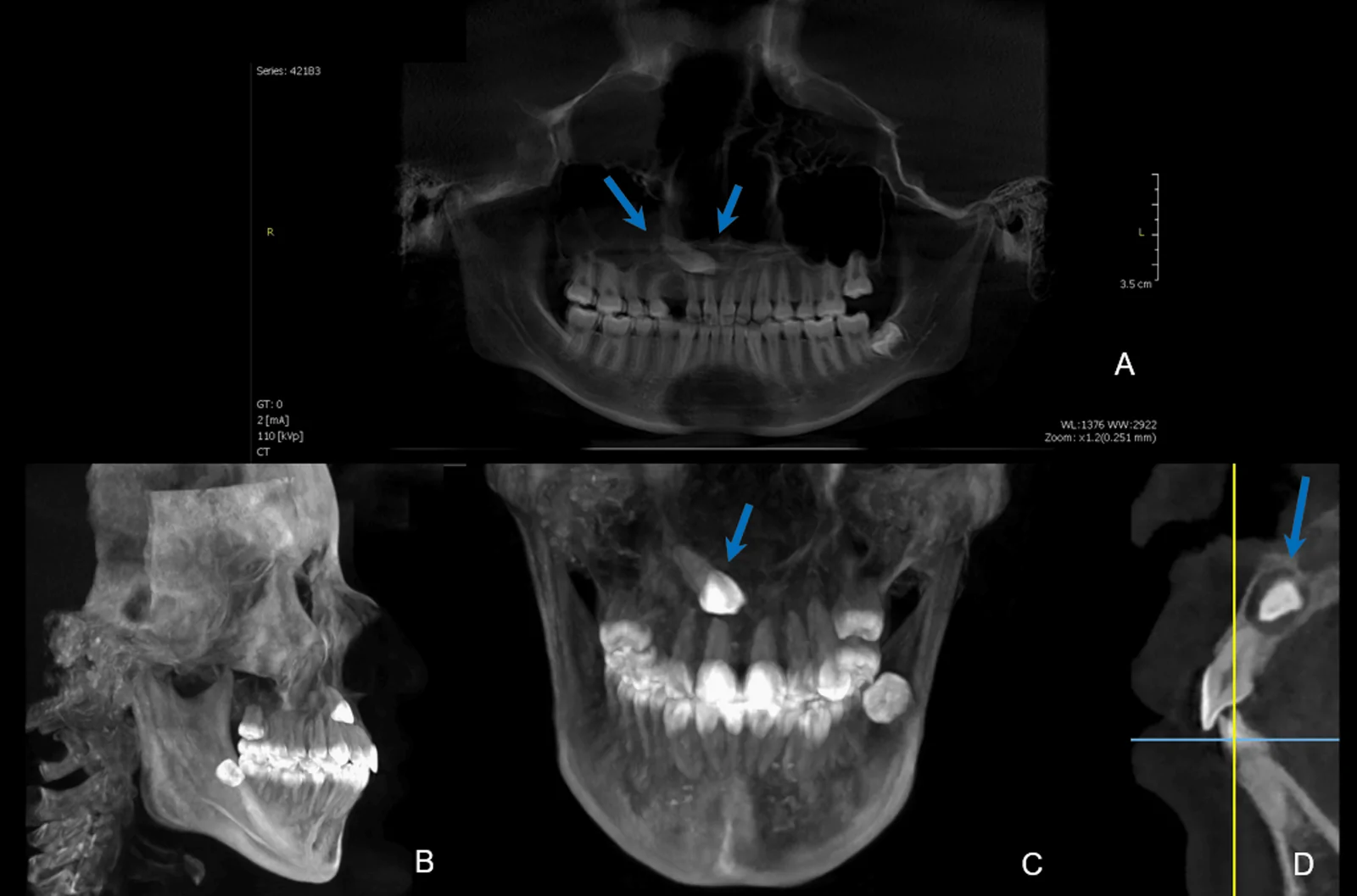
Pre-treatment CBCT showing the severely ectopic position of the impacted maxillary canine and root dilaceration of the right maxiilary first premolar A: Frontal section, B: Lateral view, C: Frontal section depicting the maxillary canine positioned near the floor of the maxillary sinus, D: Sagittal section of the central incisor (#11) region, illustrating the crown of the retained canine located above the incisor apex, CBCT: Cone-beam computed tomography The blue arrows highlight the dilacerated first premolar (#14) and the retained maxillary canine (#13).

A pre-treatment CBCT showed the right maxillary canine in a severely ectopic position, located in close proximity to the floor of the maxillary sinus, with a markedly unfavorable long axis relative to the occlusal plane. Root dilaceration of the right maxillary first premolar was also identified. No cystic or other associated pathologic lesion was observed on imaging (Figure 3).

Based on these findings, an interdisciplinary orthodontic-surgical assessment concluded that conventional orthodontic traction was not feasible. When mapped onto recognized radiographic predictors of traction difficulty, the impaction fell into the least favorable category: the crown occupied an unfavorable mesiodistal sector overlapping the adjacent roots, the angulation to the midline was severe, and the vertical position was high, adjacent to the sinus floor. Four anatomic and biomechanical considerations supported the decision: (i) proximity of the crown to the maxillary sinus floor, which severely restricted the available traction trajectory; (ii) a markedly deviated longitudinal axis requiring a complex and prolonged force vector through dense bone; (iii) root dilaceration of the adjacent first premolar, increasing the risk of iatrogenic resorption during canine movement; and (iv) an overall configuration carrying a high probability of ankylosis if traction were attempted. Taken together, these features indicated an unfavorable prognosis for the impacted canine, and extraction was therefore selected over surgical exposure and traction.

**Figure 4:**
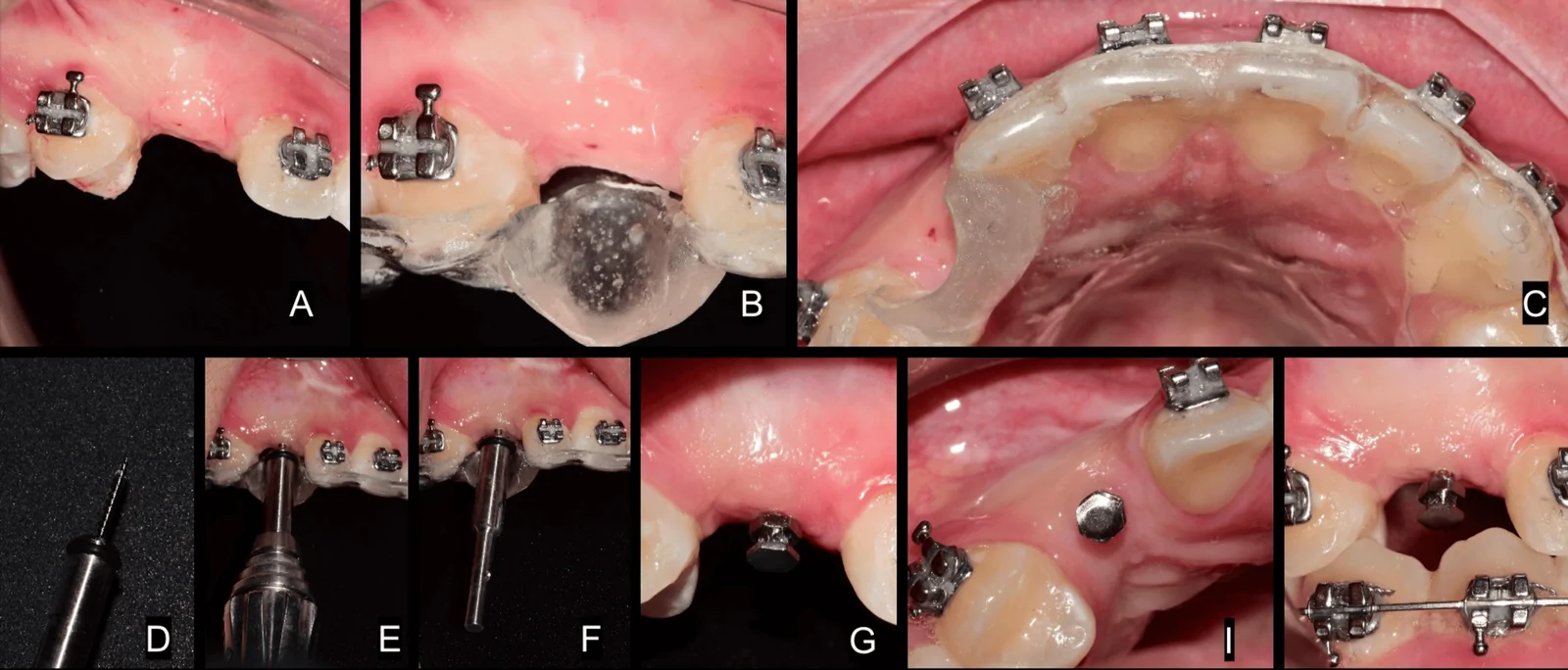
Surgical guide and microscrew placement A: Right maxillary edentulous site, B: Frontal view of the surgical guide, C: Occlusal view of the surgical guide, D: Titanium microscrew, E: Manual placement of the microscrew, F: Clinical verification of primary stability, G: Frontal view of the placed microscrew, H: Occlusal view of the placed microscrew, I: Occlusal view

For replacement of the extracted canine, a removable prosthesis, a resin-bonded restoration, orthodontic space closure, and autotransplantation were considered. Each was judged less suitable in this patient because of her age, the need to preserve intact adjacent teeth, and the requirement to maintain the space until skeletal maturity. Because the patient was still undergoing active craniofacial growth, placement of a definitive osseointegrated implant was not considered appropriate at this stage. After extraction, orthodontic space recovery was performed using conventional fixed appliances. Approximately 8 mm of space was opened over four months, and clinical stability of the recovered space was confirmed before the restorative-surgical phase was initiated. A customized surgical guide was designed and fabricated to optimize 3D positional accuracy during placement (Figure 4).

Under local infiltrative anesthesia, the microscrew was placed manually using a minimally invasive transmucosal approach, without flap elevation, with the assistance of the surgical guide. Adequate primary clinical stability was confirmed by manual verification of resistance to counter-rotation at the time of insertion. Formal insertion torque measurement using a calibrated device was not performed, in line with routine practice for orthodontic microscrew placement.

**Figure 5:**
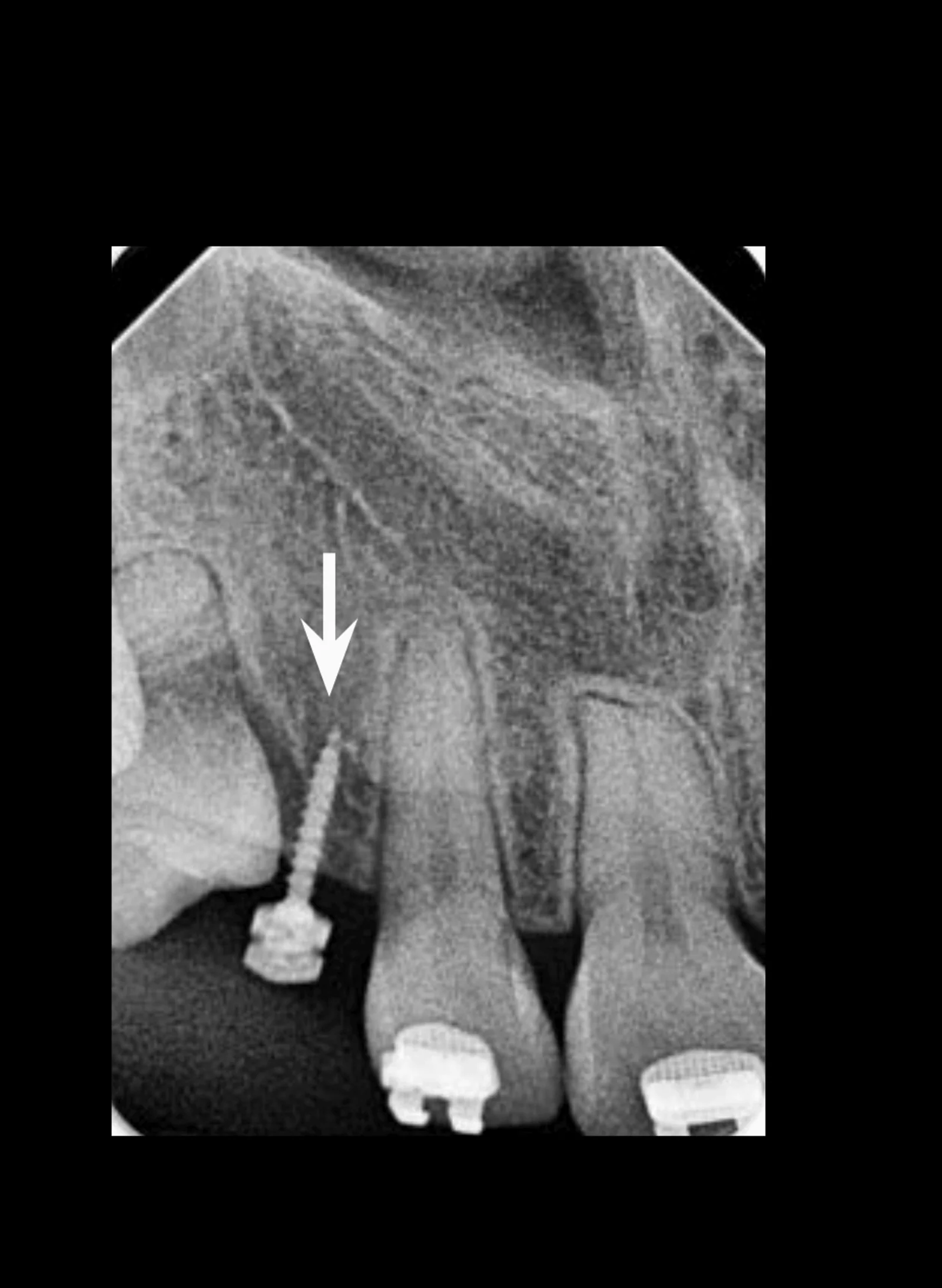
Intraoperative periapical radiograph The radiograph confirmed the position and trajectory of the microscrew relative to the adjacent root structures. The arrow indicates the microscrew placed in a parallel orientation.

**Figure 6:**
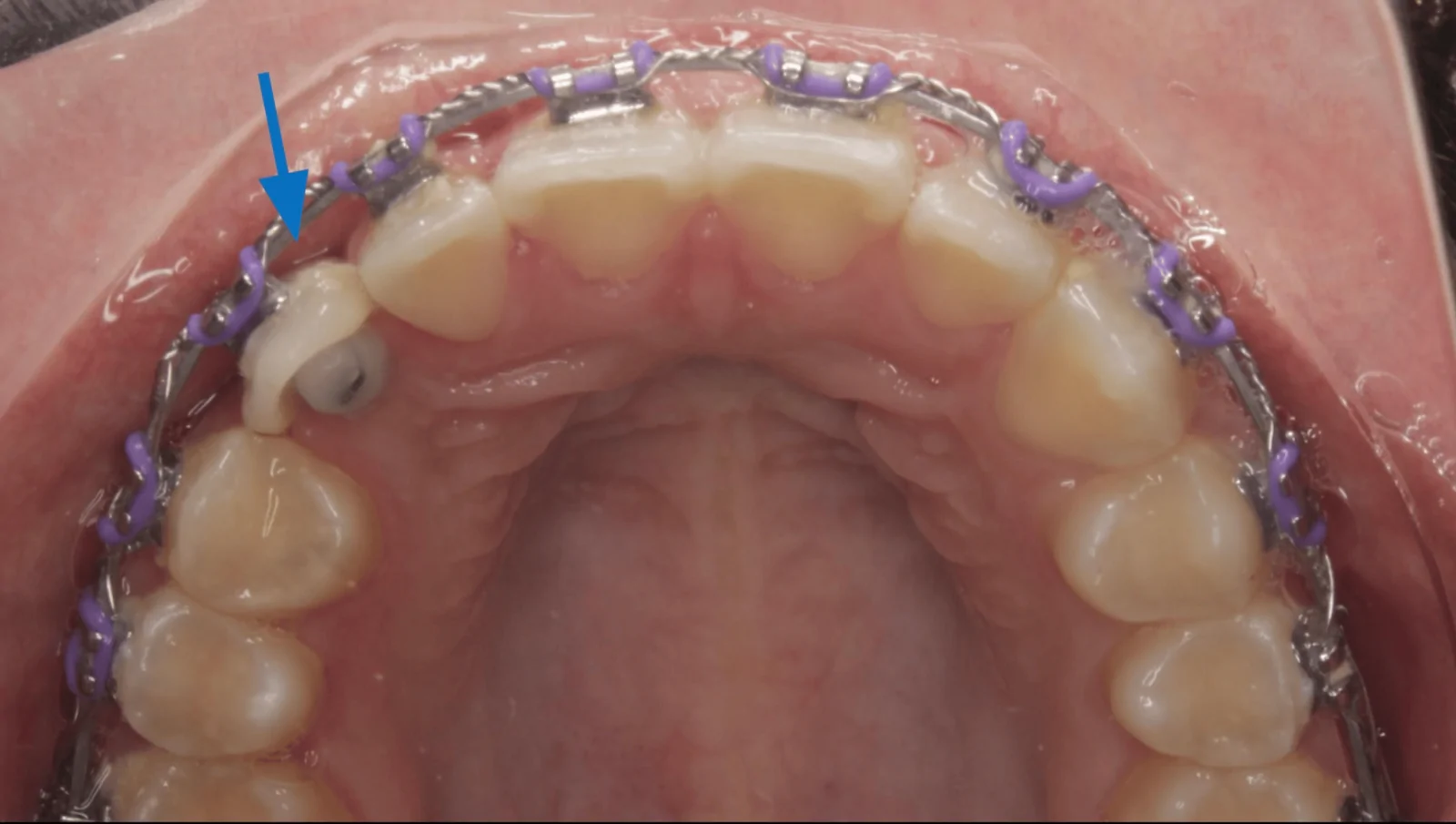
Acrylic resin provisional cover placed immediately after microscrew insertion to provide initial esthetic coverage The arrow indicates the postoperative placement of the microscrew in a parallel orientation.

**Figure 7:**
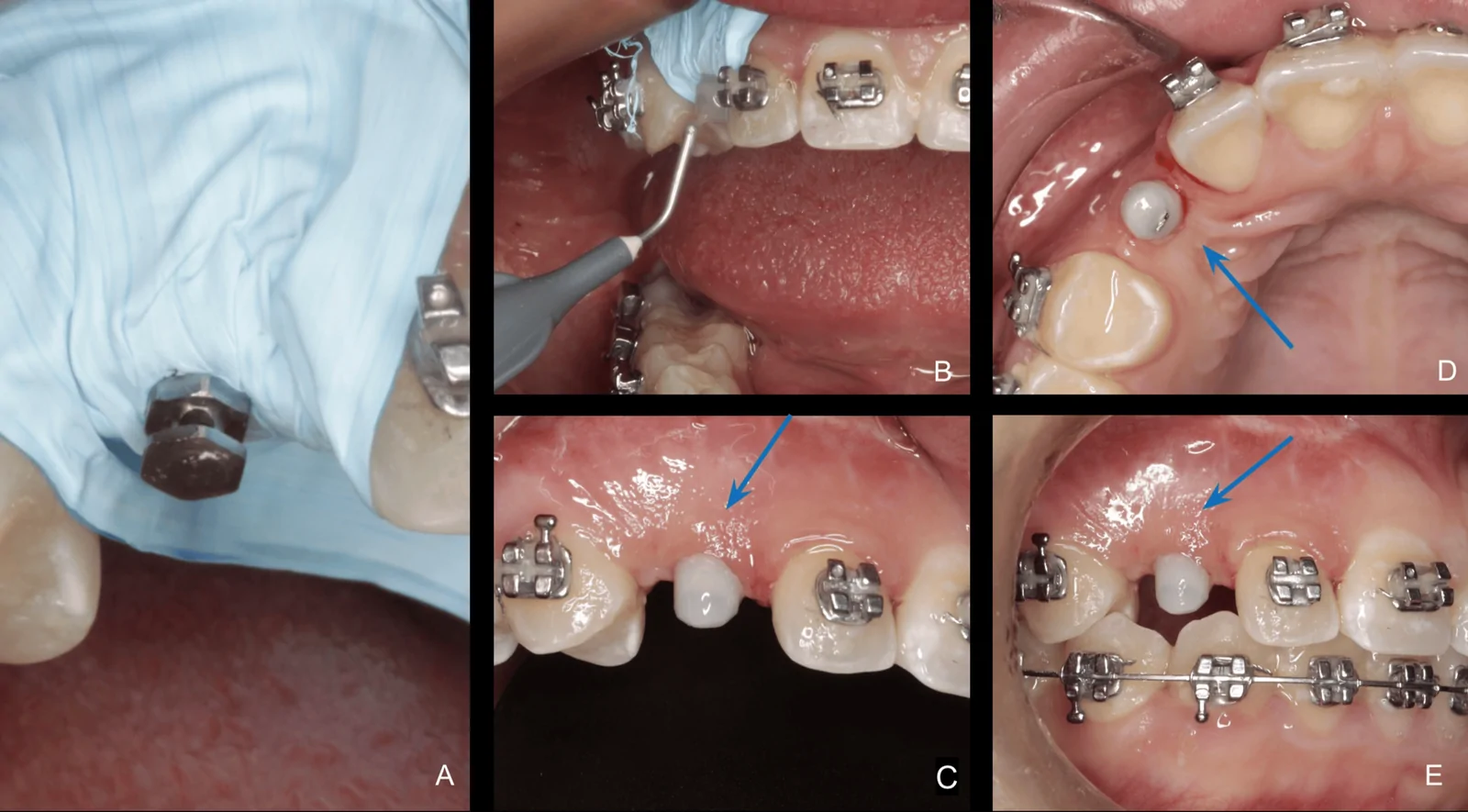
Shaping of the prosthetic abutment A: abutment isolation, B: Shaping with light-curing flowable resin, C: Frontal view of the prosthetic abutment, D: Occlusal view of the abutment. The blue arrows indicate the abutment shaping over the microscrew.

An intraoperative periapical radiograph was obtained to confirm the position and trajectory of the microscrew relative to the adjacent root structures (Figure 5). Immediately after microscrew insertion, an acrylic resin provisional cover was placed directly over the microscrew head to provide initial esthetic coverage (Figure 6). A prosthetic abutment was then shaped over the microscrew head with light-cured flowable resin (Figure 7).

**Figure 8:**
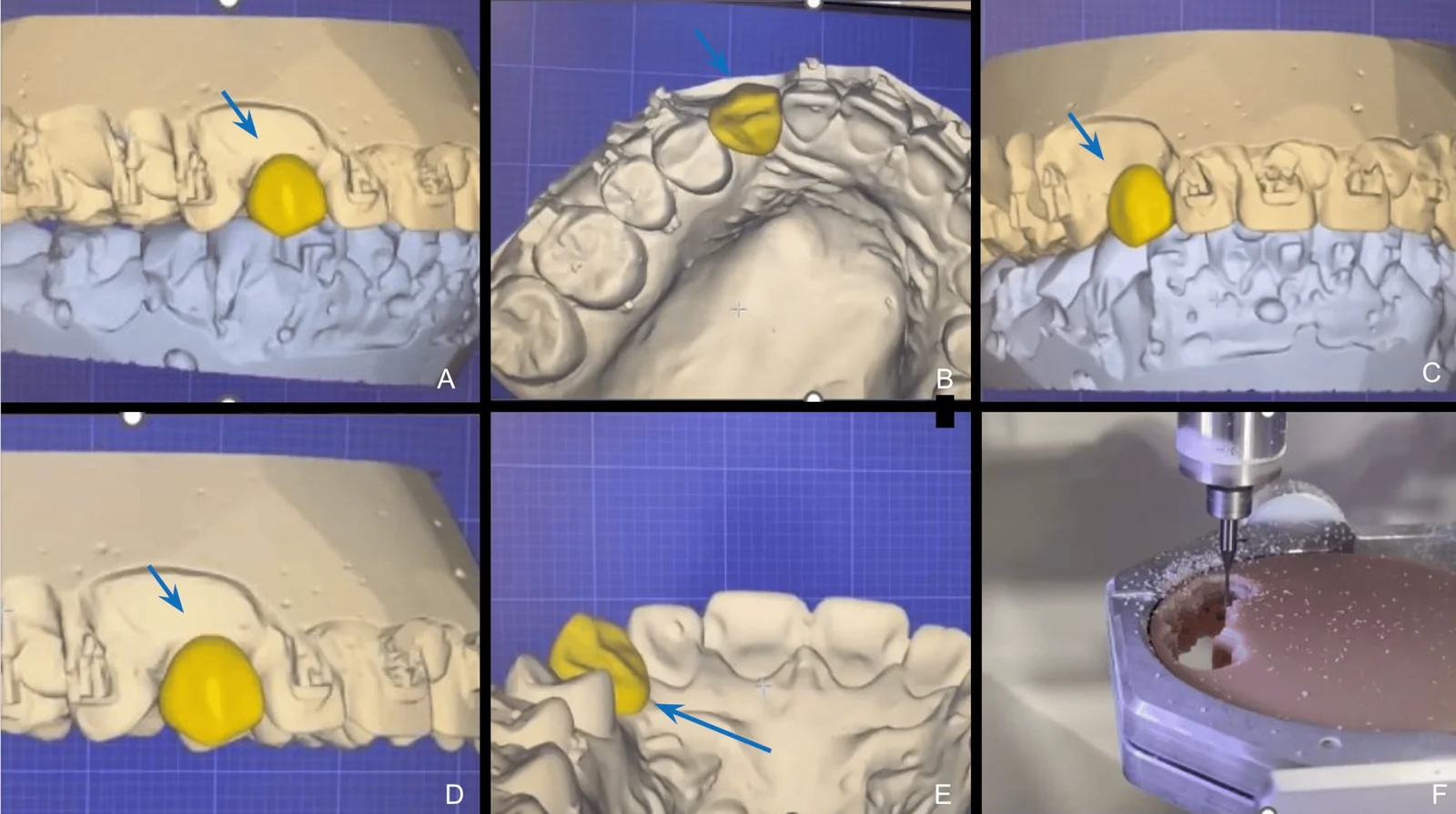
The CAD/CAM design and fabrication of the provisional polymethyl methacrylate crown A: Right lateral 3D design in occlusion, B: Occlusal 3D design, C: Frontal design in occlusion, D: Right lateral view, E: Posteroanterior view, F: 3D printing of the provisional crown, CAD/CAM: Computer-aided design and computer-aided manufacturing The CAD was performed using licensed ExoCAD software (Align Technology, Tempe, AZ, USA).

Subsequently, a definitive provisional polymethyl methacrylate crown was digitally designed through a computer-aided design and computer-aided manufacturing (CAD/CAM) workflow and produced by 3D printing (Figure 8). The crown was adjusted intraorally to ensure passive interproximal contact and the absence of occlusal contact in maximum intercuspation and during all excursive movements, in line with a non-functional immediate loading protocol.

**Figure 9:**
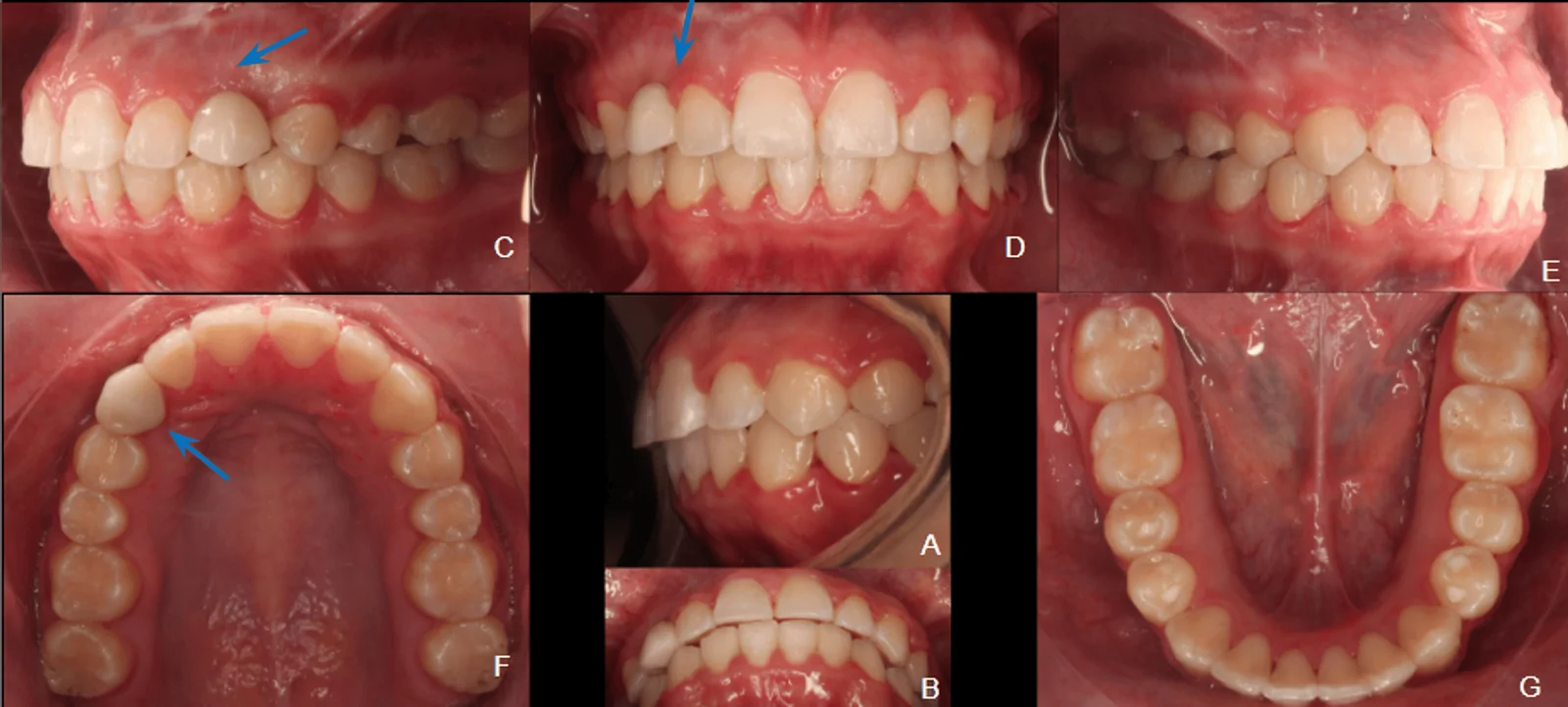
Follow-up clinical photographs showing stable occlusion and satisfactory esthetic integration at 12 months A: Overbite, B: Overjet, C: Frontal view, D: Left lateral view, E: Maxillary occlusal view, F and G: Mandibular occlusal view. The blue arrows indicate the provisional restoration at the canine site (#13).

The patient was followed clinically and radiographically for 12 months after placement of the orthodontic microscrew and the provisional restoration. Clinically, the device remained stable throughout the follow-up period, with no detectable mobility on manual evaluation at any control visit. The soft tissues surrounding the microscrew remained healthy, with no signs of inflammation, suppuration, or patient-reported symptoms. The provisional crown maintained its position without debonding, fracture, or other mechanical complications. The restoration also preserved the mesiodistal dimension of the space and provided satisfactory esthetic integration within the anterior maxillary segment (Figure 9).

**Figure 10:**
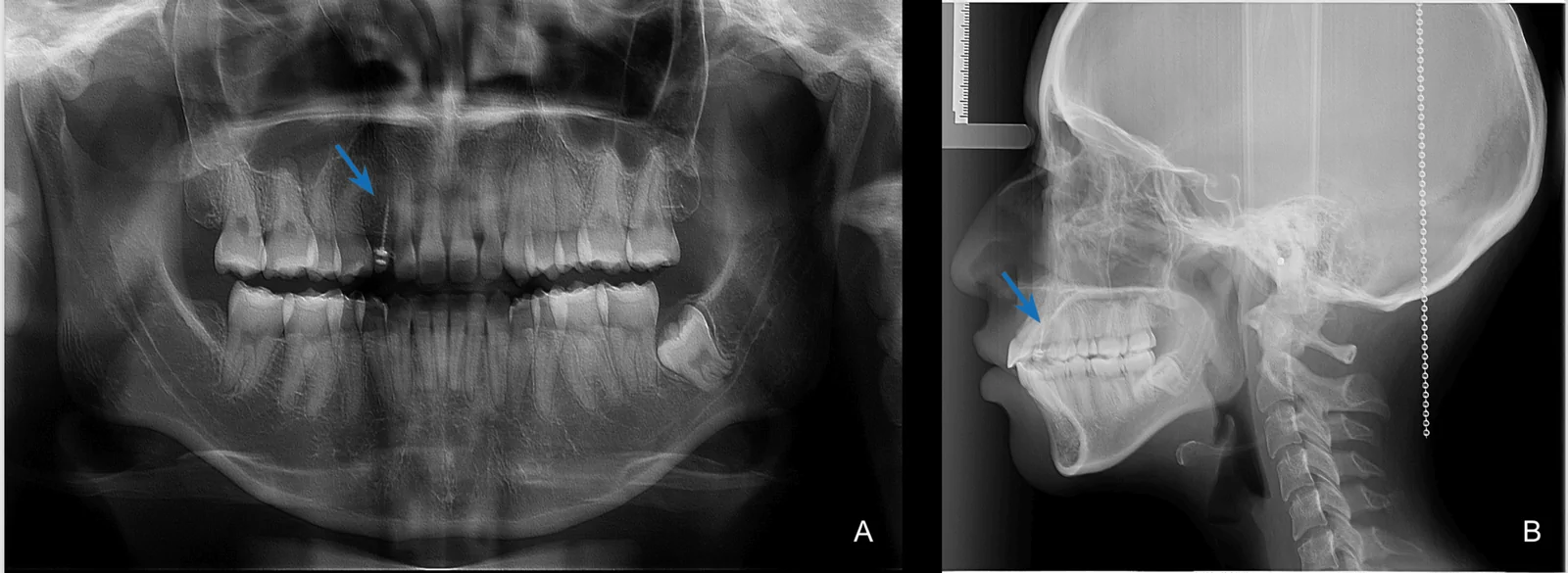
Panoramic and lateral cephalometric radiographs at 12 months confirming positional stability of the microscrew A: Final panoramic radiograph, B: Lateral cephalometric radiograph The blue arrows indicate the microscrew placed in the edentulous space of the retained canine (#13).

Objective parameters recorded at each control visit (one week; and one, three, six, nine, and 12 months) included device mobility (assessed manually and was absent at all visits), peri-screw soft-tissue status (no inflammation, suppuration, or probing-detectable pathology), occlusal clearance in maximum intercuspation and excursions (maintained at all visits), restoration integrity (no debonding or fracture), and radiographic positional stability of the microscrew (no displacement). Occlusal assessment at each follow-up visit confirmed the absence of functional loading on the provisional crown, with clearance maintained in maximum intercuspation and during lateral and protrusive excursions. No occlusal interference associated with the restoration was detected during the observation period. Panoramic and lateral cephalometric radiographs obtained at the end of the follow-up showed maintenance of the microscrew position, with no apparent displacement or radiographic signs suggestive of failure (Figure 10). No adverse radiographic changes were observed in the adjacent dental structures.

**Figure 11:**
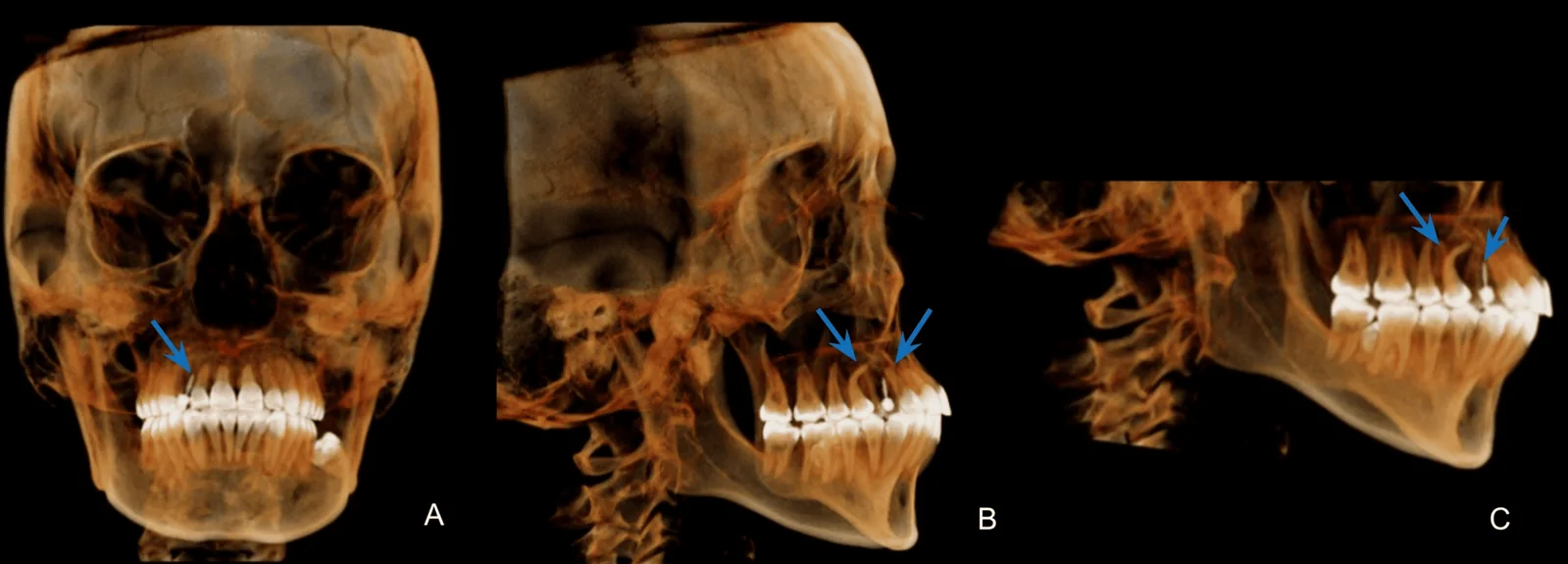
The 12-month follow-up CBCT of the treated site A: 3D frontal section, B: 3D lateral section, C: 3D view of the lower third of the maxilla at the treated site, CBCT: Cone-beam computed tomography The blue arrows indicate the microscrew in the edentulous space of the retained canine (#13) and the dilaceration of the first premolar (#14).

A control CBCT acquired 12 months after placement was reviewed for qualitative evaluation of the treated site. No apparent radiographic changes in the alveolar contour were identified in the region corresponding to the extracted canine, and the surrounding bone showed continuity with the adjacent alveolar architecture. Because no standardized 3D volumetric measurements, image superimposition analyses, or comparative baseline assessments were performed, these tomographic observations are descriptive and qualitative in nature and should not be interpreted as objective evidence of alveolar ridge preservation (Figure 11). No biologic complications, such as mucosal overgrowth, infection, or soft tissue irritation, were recorded during the observation period. No mechanical complications related to the microscrew or the provisional restoration were identified. 

## Discussion

In the present case, several anatomic factors converged to make conventional orthodontic traction clinically unfavorable: the proximity of the canine crown to the floor of the maxillary sinus, the steep axial inclination of the impacted tooth, and the presence of root dilaceration in the adjacent first premolar. Each of these features has previously been associated with increased treatment difficulty and a higher likelihood of ankylosis when traction is attempted [1,5,6]. In combination, they supported the indication for extraction over surgical exposure as the most appropriate clinical decision.

The choice of a transitional strategy in a growing patient is governed by four requirements: space maintenance, preservation of alveolar volume, protection of adjacent teeth, and deferral of definitive implant therapy until skeletal maturity. Removable prostheses satisfy none of the biomechanical requirements and are poorly tolerated at this age; resin-bonded restorations preserve space and esthetics but commit sound adjacent teeth; orthodontic space closure depends on the occlusion and sacrifices the canine relationship; and autotransplantation, a legitimate option in selected cases [7], is technique-sensitive and constrained by donor root development. A microscrew-supported provisional was selected because, uniquely among these options, it preserves both the space and the integrity of the neighboring teeth while remaining reversible.

The functional status of the canine site also shapes the loading protocol. In a natural dentition, the canine bears substantial lateral load during guidance; a mechanically retained microscrew cannot safely assume this role. Delivering the provisional crown out of occlusion in both maximum intercuspation and all excursive movements was therefore a deliberate measure to shield the microscrew from lateral and shearing forces that could compromise its stability. The uneventful 12-month course is consistent with this rationale, although it does not, by itself, establish the durability of the approach.

In skeletally immature patients, the rehabilitative phase that follows extraction is constrained by ongoing craniofacial growth. Space must be maintained, the alveolar ridge must be preserved as far as possible, and the integrity of adjacent teeth must be respected, while definitive implant placement is deferred. Osseointegrated implants placed before skeletal maturity behave as ankylosed units and may become progressively infraoccluded as alveolar development continues [8,9]. Among the available transitional options, removable prostheses are poorly tolerated by adolescents and do not transmit physiologic load to bone, while adhesive bridges require the commitment of otherwise sound adjacent teeth to the prosthesis. The clinical rationale for considering an orthodontic microscrew in this setting is that it preserves both the available space and the integrity of the neighboring teeth during the waiting period.

Most published experience with microscrew-supported transitional restorations in growing patients concerns the maxillary lateral incisor region, with favorable short-term outcomes reported under non-functional loading protocols [11-13]. The canine site differs from the lateral incisor region in two clinically relevant respects: it is subject to higher functional demands and to greater esthetic visibility. The favorable 12-month course observed in the present case suggests that the same biomechanical rationale may apply to the canine region, although the available evidence at this anatomic location remains limited and findings from a single observation cannot be extrapolated to other patients or treatment intervals.

The radiographic interpretation requires a comparable degree of caution. The 12-month CBCT did not show apparent changes in the alveolar contour at the treated site under qualitative assessment. Experimental evidence in an animal model has suggested that mechanical loading may influence bone remodeling around miniscrews [15]; however, no comparable human evidence is currently available for microscrews used exclusively as prosthetic supports. Accordingly, the maintenance of alveolar contour observed radiographically should be regarded as a hypothesis-generating observation rather than a demonstrated effect of the device.

Other reported alternatives for severely compromised maxillary canines in selected patients include autotransplantation and immediate implant placement after extraction in adults [15-17]. Both approaches may yield acceptable outcomes when properly indicated, but they are technique-sensitive and not directly applicable to a growing adolescent patient with incomplete skeletal development.

This report has the inherent limitations of a single-case design, including the absence of a control group and the lack of comparison with alternative rehabilitation strategies. The CBCT follow-up was qualitative, since no baseline superimposition or volumetric measurements were performed; consequently, any dimensional change at the treated site could not be quantified. Insertion torque was not recorded with a calibrated device. Primary stability was assessed clinically by manual verification of resistance to counter-rotation, which is consistent with routine orthodontic practice but does not provide a reproducible numerical value. Skeletal maturity was estimated on clinical grounds rather than through a standardized protocol such as the cervical vertebral maturation method. Finally, a 12-month observation period is not sufficient to address the behavior of the device as growth concludes or its long-term survival when used exclusively as a prosthetic support.

## Conclusions

In this adolescent patient, placement of a titanium orthodontic microscrew with a CAD/CAM-fabricated provisional polymethyl methacrylate crown maintained the space left by extraction of a severely ectopic maxillary canine deemed unsuitable for orthodontic traction. During a 12-month follow-up period, the device remained clinically stable, peri-implant tissues were healthy, and no biologic or mechanical complications were observed. Qualitative radiographic follow-up showed no apparent change in the alveolar contour at the treated site.

Within the limitations of a single case, an orthodontic microscrew used in this manner appears to represent a clinically feasible transitional alternative in growing patients in whom definitive rehabilitation must be postponed. Prospective studies with larger samples, standardized 3D imaging protocols, and follow-up periods extending into skeletal maturity are required to clarify the long-term dimensional and biologic behavior of this approach.
